# RUNX1 promote invasiveness in pancreatic ductal adenocarcinoma through regulating miR-93

**DOI:** 10.18632/oncotarget.20433

**Published:** 2017-08-24

**Authors:** Yin Cheng, Haiyan Yang, Yang Sun, Hongkai Zhang, Shuangni Yu, Zhaohui Lu, Jie Chen

**Affiliations:** ^1^ Department of Pathology, Peking Union Medical College Hospital, Chinese Academy of Medical Sciences and Peking Union Medical College, Beijing, China

**Keywords:** pancreatic ductal adenocarcinoma (PDAC), miR-93, RUNX1, HMGA2 (High mobility group AT-hook 2), tumor progression

## Abstract

Runt-related transcription factor 1(RUNX1), a key factor in hematopoiesis that mediates specification and homeostasis of hematopoietic stem and progenitor cells (HSPCs), is also overexpressed in several solid human cancers, and correlated with tumor progression. However, the expression and function of RUNX1 in pancreatic ductal adenocarcinoma were still unclear. Here, we show that RUNX1 is highly expressed in pancreatic adenocarcinoma tissues and knocking down of RUNX1 attenuated aggressiveness in pancreatic cell lines. Moreover, we found that RUNX1 could negatively regulate the expression of miR-93. Bioinformatics method showed that there are two binding sites in the the promotor region of miR-93 precursor and through ChIP-qPCR and firefly luciferase reporter assay, we vertified that these two binding sites each have transcriptive activity in one pancreatic cell lines. This result supported our presumption that RUNX1 regulate miR-93 through binding to the promotor region of miR-93. Besides, the expression and function of miR-93 is quite the opposite, miR-93 overexpression suppresses migration and invasiveness in pancreatic cell lines supporting that RUNX1 negatively regulated miR-93. Our findings provided evidence regarding the role of RUNX1 as an oncogene through the inhibition of miR-93. Targeting RUNX1 can be a potential therapeutic strategy in pancreatic cancer.

## INTRODUCTION

Pancreatic ductal adenocarcinoma remains to be one of the most lethal malignancies worldwide, which ranks the fourth, fourth and sixth leading cause of cancer-related deaths in the United States, European Union and China, respectively [1–3]. Because of their retroperitoneal location and no reliable early diagnostic markers, over 80% patients have been in the advanced-stage at diagnosis [4]. Unfortunately, multidisciplinary approaches in pancreatic cancer management have yet to result in a meaningful increase of 5-year survival [4, 5]. As the majority of pancreatic cancer (85% of which are ductal adenocarcinoma) present aggressive features, it is vital to understand more about the basis for pancreatic ductal adenocarcinoma (PDAC) progression and to develop more effective treatment for this deadly disease.

The RUNX (Runt-related transcription factor) family of genes are significant regulators controlling pancreatic cancer progression. It has been reported that RUNX-2 was highly expressed in pancreatic tumor cells, PanIN lesions and tumour-associated fibroblasts/stellate cells, but showed weak to absent expression in normal pancreatic tissues and RUNX2 has the potential to regulate expression of extracellular matrix modulators SPARC and MMP1, thus influencing tumor microenvironment [6]. In addition, Runx3 plays dual roles leading to enhanced migration and metastasis ability while showing reduced proliferation in PDAC [7, 8]. However, the role RUNX1 played in the carcinogenesis or progression of pancreatic cancer is largely unknown. Altered expression of RUNX1 is associated with many other cancers. Recently, mutations of transcription factor *CBFB* and deletions of *RUNX1* causing Runx1/Cbfb complex loss of function in breast cancer has been identified by whole-genome sequencing. RUNX1 mutation in breast cancer can predict poor outcome, all indicating that defective RUNX1 function might also act as an important factor in epithelial tumors, not only in acute myeloid leukemia [9, 10]. In another study, up-regulation of RUNX1 in endometrioid carcinoma (EEC) was correlated with the initial myometrial infiltration [11]. Similarly, upregulated RUNX1 was involved in the establishment of an orthotopic endometrial cancer mouse metastases model [12]. RUNX1 was also over expressed in human and mouse neurofibroma-initiating cells with no neurofibromatosis type 1(*Nf1*) context [13]. Those studies suggest that RUNX1 contributes to cancer progression. Although the biological effects of RUNX1 are clearly established in multiple malignancies, the underlying molecular mechanisms underlie remain unclear.

Here, we have defined an innovative oncogenic pathway downstream of RUNX1 involving miR-93-dependent regulation of HMGA2 in PDAC. Over-expressed RUNX1 was related to poor prognosis. The mechanism mediating this oncogenic effect was that RUNX1 negatively regulated miR-93 by directly interacting with its promoter; subsequently miR-93 induced degradation of miR-93 target gene HMGA2 was abrogated. Further, we showed that overexpression of miR-93 or knocking down of HMGA2 both can decrease the invasive ability of pancreatic cancer. Together, these findings defined a novel potential axis activated by the upregulated RUNX1 leading to pancreatic cancer invasion that may help the design of future clinical studies for this devastating disease as well as other diseases with aberrant levels of RUNX1.

## RESULTS

### RUNX1 is upregulated in PDAC and high RUNX1 expression showed reduced suvival time

**Figure 1:**
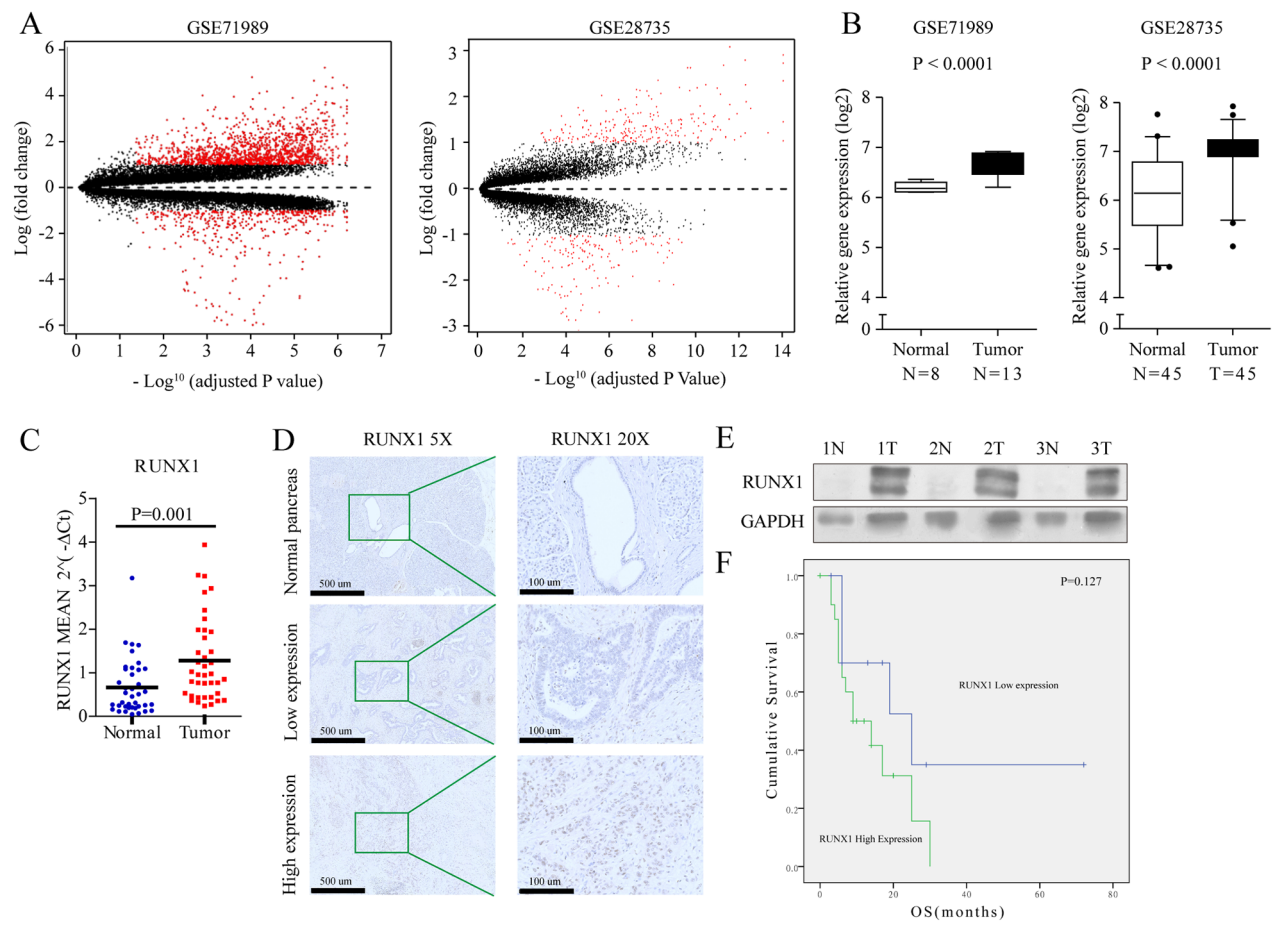
**(A)** Volcano plot showing relationship between magnitude of gene expression change (log2 of fold-change; *y*-axis) and statistical significance of this change [-log10 of adjusted p value; *x*-axis] in a comparison of tumors and adjacent non-tumor tissues in GSE71989 (left) and GSE28735 (right) cohorts. Red points represent differentially expressed genes (with cut-off FDR < 0.05) with magnitude of change ≥2. **(B)** Gene expression of RUNX1 in two independent cohorts of PDAC. RUNX1 is increased in tumors compared with non-tumor tissues in GSE71989 (left) and GSE28735 (right) cohorts. Box plots represent gene expression level with relative intensity (log2) of microarray data. Bars indicate median value. Student t test. **(C)** Average relative RUNX1 expression level in PDAC compared with that in normal tissues. Expression of RUNX1 was measured by qRT-PCR and normalized by GADPH. **(D)** Immunohistochemical staining for RUNX1 protein in normal pancreas and PDAC tissues, brown granule in nucleus showed low or high expressed RUNX1 with lower magnification images (5×, left) and its expanded views (20×, right). **(E)** Protein expression in 3 paired PDAC and the adjacent normal pancrea tissues. RUNX1 protein expression level was determined by immunoblotting with GADPH as a control. **(F)** Kaplan-Meier curves for OS in PDAC patients with RUNX1 expession. Gene expression of RUNX1 is divided into high and low expression groups using a median cutoff. Log-rank P value is indicated in the graphs.

To screen pancreatic cancer gene expression profiles, we selected two qualified gene expression microarray datasets (GSE71989 and GSE28735), and identified genes that were differentially expressed in pancreatic tumors and non-tumor tissues (P<0.01) in both independent cohorts (Figure 1A). Interestingly, RUNX1 was more highly expressed in pancreatic tumors chips (Figure 1B), but its expression in solid tumors is still unkown, especially in pancreatic adenocarcinoma (PDAC).

To further validate these findings, quantitative PCR was done in 76 pancreatic fresh samples, including 39 cancers and 37 matched adjacent normal tissues or seperate normal tissues. In this analysis, 61.5% (24/39) of pancreatic cancer samples had at least a 1.5-fold increase in RUNX1 mRNA expression levels relative to normal samples (P=0.001) (Figure 1C).

To evaluate whether RUNX1 protein expression is also increased in pancreatic cancer, immunohistochemistry for RUNX1 was done in 39 PDAC cases, each including tumor and normal tissues in the same slide. Of the 39 pancreatic cancer tissues examined, the longest had 3 years of clinical follow-up. In normal pancreatic tissues, RUNX1 expression was detected weak and focal. None of the normal pancreatic tissues had a score higher than 1+ (Figure 1D). The majority (26/39, 66.7%) of pancreatic cancers received scores of 2+ or above, while a minority (13/39, 33.3%) of the pancreatic cancers had low level of RUNX1 expression (Figure 1D). High level of RUNX1 expression showed no correlation with any clinical-pathological features (Supplementary Table 2). Increased RUNX1 protein expression (>= 2+) was found to increase the hazard ratio (HR) for death (HR=3.177, 0.083-1.126, P =0.075). Besides, Age and positive margin also increase the HR for death (HR=2.851, 0.099-1.187, P=0.091) and (HR=1.989, 0.106-1.441, P =0.158), respectively (Supplementary Table 3). Western blot of three paired cancers and matched adjacent normal tissues also showed a higher level of RUNX1 expression relative to normal pancreatic tissues (Figure 1E).

The relationship between RUNX1 expression and clinical outcome was then assessed by looking at over-all survival in the 39 patients. Of 39 patients, the follow-up time was between 3 to 72 months. 17 had died by the last follow-up and the median survival time was 9 months. Survival analyses showed difference between RUNX1 low expression (mean of 34.7 months – 95% confidence interval, 13.92-55.48 months) and RUNX1 high expression tumor cells (mean of 14.3 months – 95% confidence interval, 9.43-19.23 months) (Figure 1F). High RUNX1 level has a shorter overall survival time for patients (Figure 1F).

### RUNX1 promotes PDAC cells invasion

**Figure 2:**
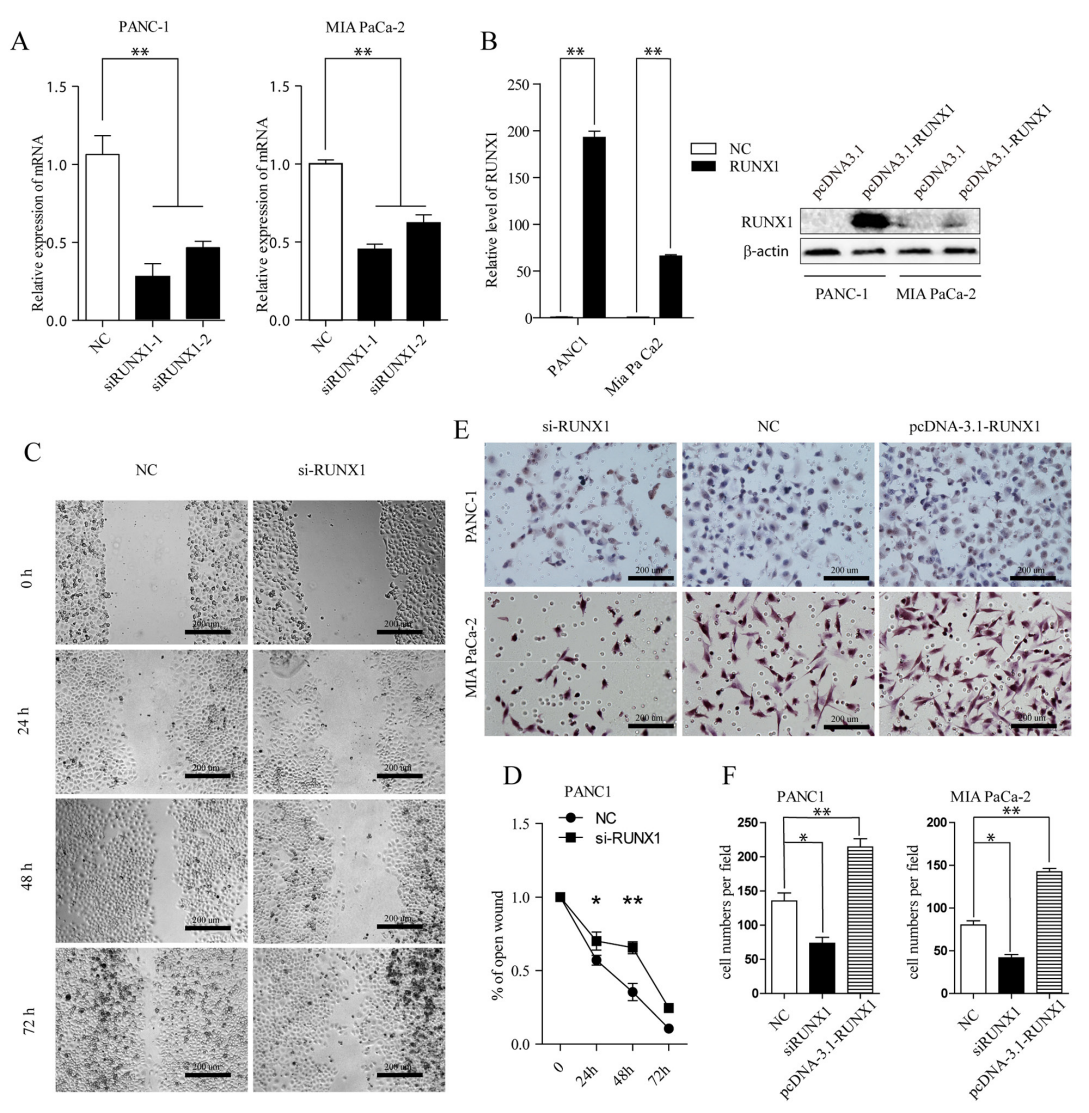
**(A)** RUNX1 knocking down efficiency was measured by RT-qPCR assay in two cells (siRUNX1-1 and siRUNX1-2 are different siRNA fragments). ^**^ P < 0.01. **(B)** Overexpression of RUNX1. Cells were transfected with pcDNA3.1-RUNX1 or empty vector pcDNA3.1. Overexpression of RUNX1 was confirmed by RT-qPCR (Left) and Western blotting (Right) in two cells, respectively. ^**^P < 0.01, t test. **(C, D)** Wound healing assay. PANC-1 cell was transfected with Negative Control (NC) and si-RUNX1. Optical images of wounded cell monolayers were taken after 0 h, 24 h, 48 h, and 72h. The remaining distance was calculated as a percentage of the initial wound area. ^*^P<0.05 or ^**^P<0.01. **(E, F)** Two pancreatic cell lines transfected with NC, si-RUNX1 and pcDNA3.1-RUNX1 cells were collected and induced to invade through Matrigel-coated transwell membranes. The numbers of migrated cells were counted. ^**^ P < 0.01.

Given these clinical links, we further investigated the biological effect of RUNX1 on pancreatic cancer invasion. We first knocked down RUNX1 through transfecting pancreatic cell lines (PANC-1, MIA PaCa-2) with two different siRNAs designed for RUNX1 (siRUNX1 I and II), both of them could efficiently decrease the endogenous expression of RUNX1 mRNA (Figure 2A). Firstly, wound healing cell migration assay revealed the move ability of PANC-1 cells was markedly decreased following downregulation of RUNX1 (Figure 2C–2D). Consistently, the invasive capacity was also drastically reduced following RUNX1 downregulation using transwell assay (Figure 2E–2F). MIA PaCa -2 cells were abandoned of this wound healing assay owing to its limited mobility.

In contrast, we further examined the role of RUNX1 by overexpression RUNX1 level using a pcDNA3.1-RUNX1 expression vector, in two pancreatic tumor cells (Figure 2B). 48 h later after transfection, RUNX1 mRNA level in both cells was noticeably increased; the protein expression of RUNX1 in both cell lines was also significantly upregulated compared with empty vector groups (Figure 2B). Here, we examined the role of RUNX1 gain of function using transwell system in both cell lines. Transwell invasive assays showed that RUNX1 upregulation promoted cells invasion (Figure 2E–2F). Taken together, these results indicate that RUNX1 may contribute to pancreatic cancer progression through regulating pancreatic cells migration and invasion.

### RUNX1 negatively regulated miR-93

Our previous results indicate that microRNAs could trigger key driver genes’ dysregulation such as K-ras in pancreatic ductal adenocarcinoma (PDAC), thus contributing to pancreatic cancer suppression [15–17]; microRNAs were also reported to be regulated by transcription factor (TF) because these microRNAs have binding sites on their promotor [18]. Since RUNX1 was a transcription factor, we want to dig deep on the mechanisms on how RUNX1 works as an oncogene.

**Figure 3:**
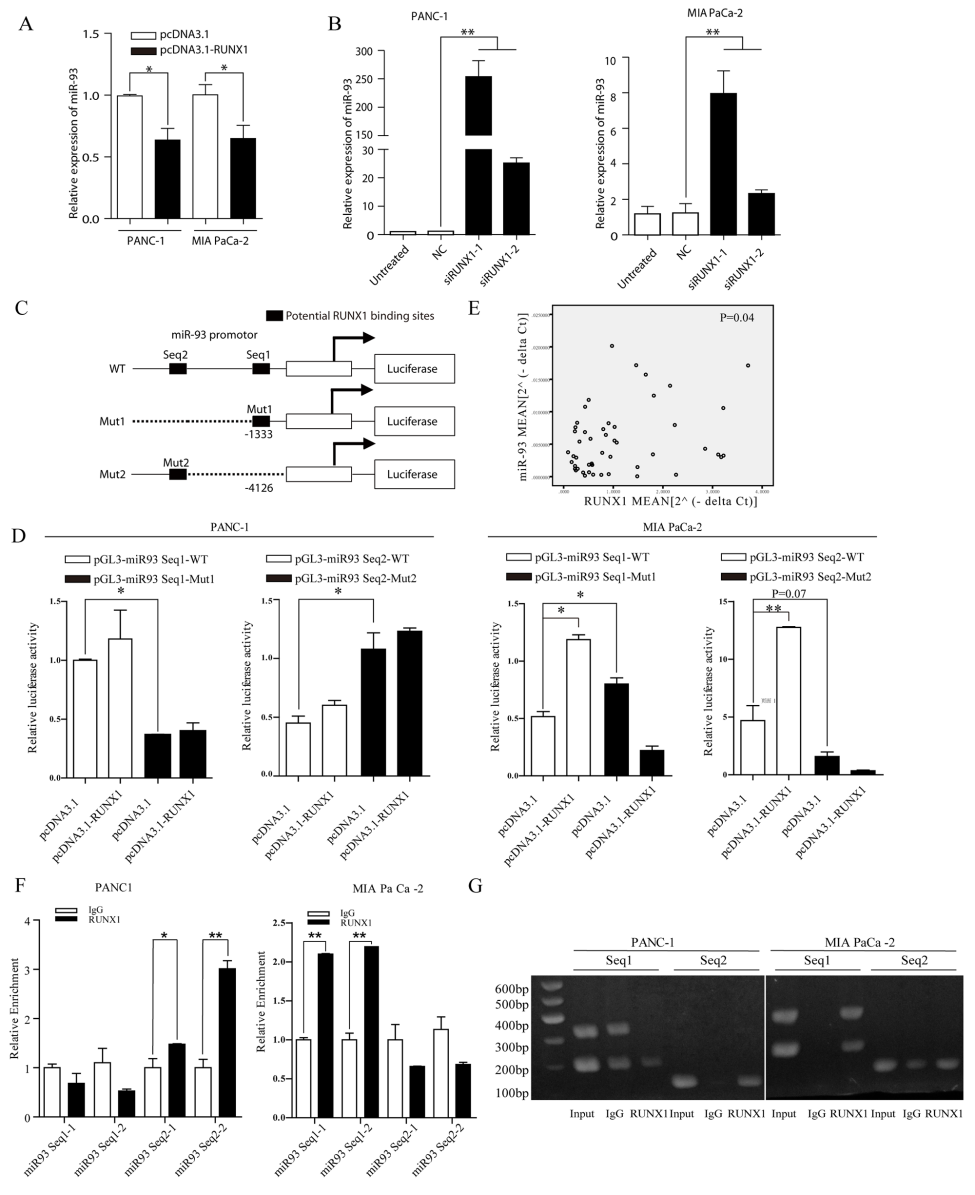
**(A)** miR-93 expression decreased in pcDNA3. 1-RUNX1 transefected PANC-1 and MIA PaCa-2 cells, compared with pcDNA3.1 group. Data were normalized by U6. ^*^P<0.05. **(B)** Relative miR-93 expression level measured by RT-qPCR. Two cells were separately transfected with NC and two RUNX1 siRNA (siRUNX1-1, siRUNX1-2). Results were normalized by U6. ^**^ P < 0.01. **(C)** Schematic diagram of the pGL3 luciferase reporter constructs containing miR-93 precurser and upstream two potential RUNX1 binding sites (-1333 and -4126). The black boxes indicate the two predicted RUNX1 binding site in the promoter region of miR-93, respectively. The white box indicates miR-93 precurser. Seq1 and Seq2 refer to each sequence long enough to cover binding sites for primers use or sequence insert). **(D)** The luciferase reporter constructs of the truncated miR-93 promoters and corresponding mutates were introduced into PANC-1 and MIAPaCa-2 cells pretransfected with pcDNA3.1-RUNX1 or empty. Luciferase activity was measured 48 h post transfection. **(E)** Expression of miR-93 was positively correlated with that of RUNX1 (P=0.04) in clinical samples. **(F)** Fold-enrichment of RUNX1 binding at the miR-93 promoter relative to background in PANC-1 and MIA PaCa-2 cells was measured by RT-qPCR. Normalized to GAPDH, results were adjusted as n-fold compared to IgG. Data are presented as mean ± SEM. ^**^ P<0.01. **(G)** Agarose gel analysis of ChIP-PCR products.

Using bioinformatics methods ChIPBase [19] and GTRD [20] and review of the literature [21], we found that there are two binding sites of the miR-93 promoter region that can potentially act as a transcript site (name as Seq1 and Seq2) for RUNX1 (Figure 3C). We first overexpressed and knock down RUNX1 in pancreatic cell lines (PANC-1, MIA PaCa -2) to see whether the miR-93 level can be altered. RT-PCR showed that when RUNX1 was knocked down, miR-93 expression was upregulated (Figure 3B). While RUNX1 was overexpressed, miR-93 expression was downregulated (Figure 3A). This result was shown in two pancreatic cell lines, suggesting a negatively regulated expression of RUNX1 and miR-93 expression in pancreatic cells.

To further validate these findings, quantitative PCR in fresh tissues was done in 24 cancers and 26 matched adjacent normal tissues including 17 paired pancreatic samples. However, RUNX1 and miR-93 expression in pancreatic cancers and corresponding normal tissues was positive correlated (P=0.04) (Figure 3E).

Dual luciferase reporter experiments was used based on inserting sequence containing binding sites and the corresponding mutated sites into pGL3-basic to test whether the two potential binding sites of the promotor region actually have transcript activity. We also designed primers covering these two binding sites for ChIP-qPCR, results showed that in PANC-1 cell lines, Seq 2 have transcript activity (Figure 3F–3G) and transcription can be suppressed compared with mutated group (Figure 3D), while in MIA PaCa -2 cell lines, Seq 1 have transcript activity (Figure 3F–3G) and transcription can be suppressed compared with mutated group (Figure 3D). Interstingly, RUNX1 overexpression group showed higher transcript activity in two cell lines, especially in MIA PaCa -2 (Figure 3D). This may due to the different binding ability of these two sites and also indicated that these two binding sites actually work in pancreatic cell lines and they may partially contribute to the interaction between RUNX1 and miR-93.

### MiR-93 suppress migration, invasion and epithelial and mesenchymal transformation in pancreatic tumor cells

**Figure 4:**
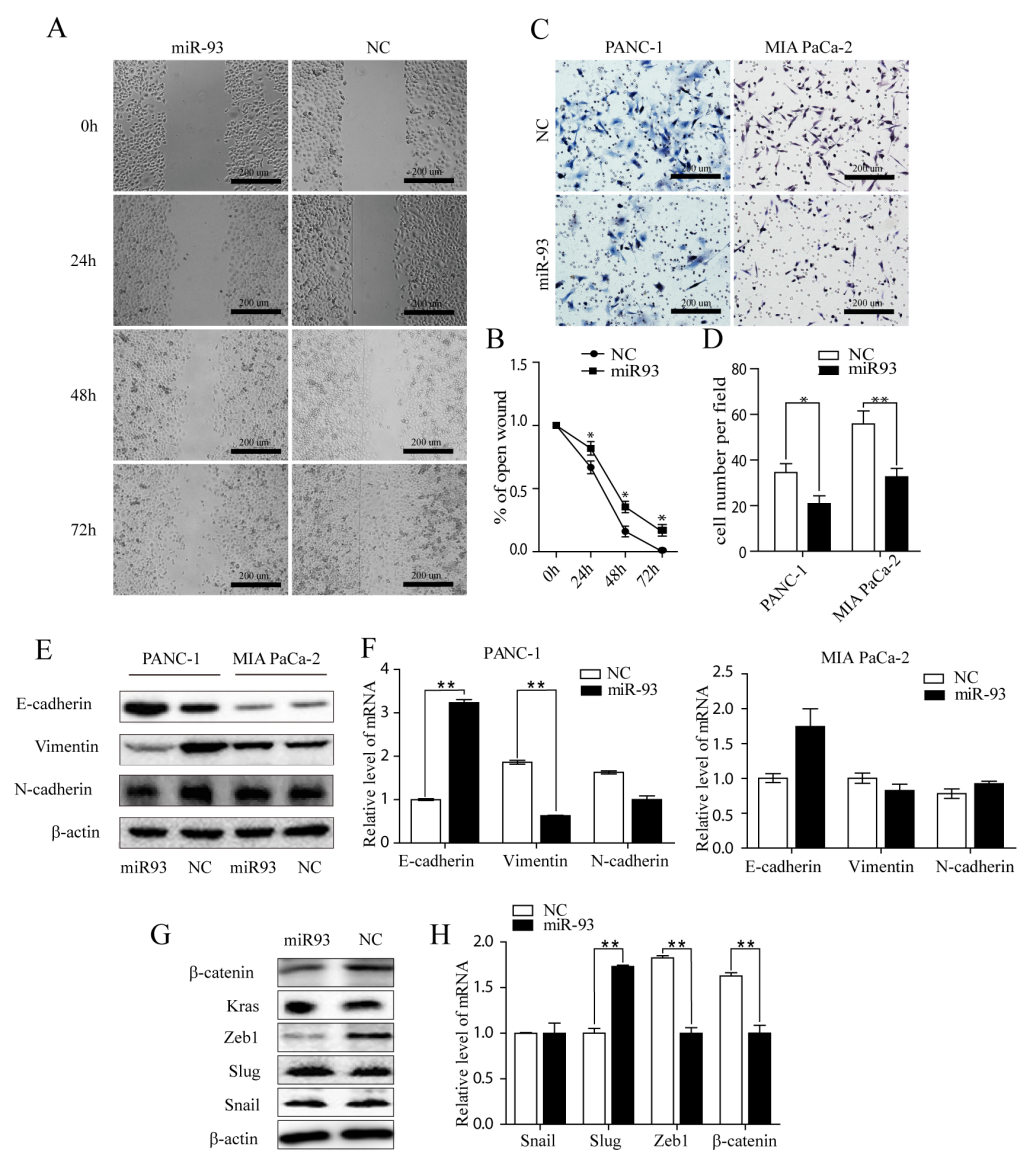
**(A, B)** Representative photos of wound healing assay of PANC-1 at 0 h, 24 h, and 48 h after wound scratch. The remaining distance was calculated as a percentage of the initial wound area. ^*^P < 0.05. **(C, D)** Representative images of cells that migrated through transwell inserts with matrigel. Data are presented as means±SEM based on three independent experiments in triplicate. **(E)** E-cadherin, vimentin and N-cadherin expression in PANC-1 and MIA PaCa-2 cells after transfecting with NC or miR-93 mimic, respectively. Western blot analysis with β-actin as the internal control. **(F)** mRNA level of EMT markers in PANC-1 and MIA PaCa-2 cells after transfecting NC or miR-93 mimic, respectively. RT-qPCR analyses were presented, separately. Data were means ± SEM of triplicates. ^*^P < 0.05, ^**^P<0.01. NC, Negative control microRNA. **(G)** Snail, Slug, Zeb1 and β-catenin, K-ras expression after transfecting NC and miR-93 mimic was detected by western blot analysis with β-actin used as the internal control. **(H)** mRNA levels of EMT regulators (Snail, Slug, Zeb1, β-catenin) in PANC-1 cells after transfecting NC and miR-93 mimic. RT-qPCR analyses were presented as means ± SD of triplicates. ^*^P< 0.05, ^**^P<0.01.

We further examined the role of MIR-93 in PDAC cells by overexpressing miR-93 using MIR-93 mimics. Wound healing assay in PANC-1and transwell invasive assays in PANC-1and MIA PaCa-2 showed that enhanced expression of MIR-93 suppressed cell invasion in both PANC-1 and MIA PaCa-2 PDAC cells (Figure 4B and 4D).

Epithelial-mesenchymal transition (EMT) is an early event migration of cancer, also indicating an invasive potential of tumor cells, here, we found that in PANC-1 cell lines, epithelial marker E-cadherin was greatly increased and mesenchymal markers vimentin, N-cadherin were drasticlly decreased after MIR-93 transfection on both protein and mRNA level (Figure 4E–4F), suggesting an EMT suppression status. However, MIA PaCa-2 cell lines were not as susceptible and didn’t change as much (Figure 4E–4F). This may be due to the indolent behaviour of MIA PaCa-2, which were spindle and morphologically showed a mesenchymal phenotype. They lack adhesion and showed a lower E-cadherin expression (Figure 4E) than PANC-1 cell lines.

Other EMT related transcription factor in PANC-1 after MIR-93 transfection were also tested. β-catenin and ZEB1 expession decreased, while snail, slug didn’t show obvious changes. (Figure 4G–4H). We also added K-ras, it doesn’t change either.

Taken together, these results all indicate that overexpression of MIR-93 suppress migration and invasiveness of PDAC cells.

### MiR-93 target HMGA2

**Figure 5:**
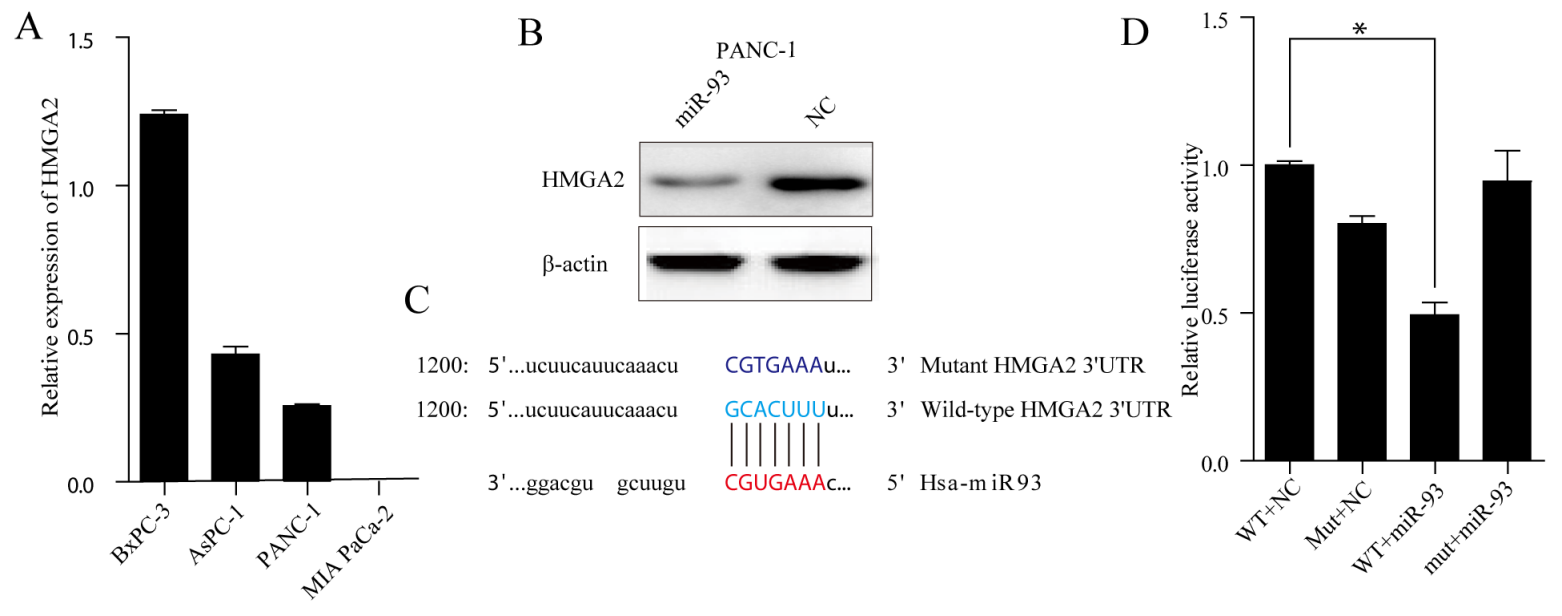
**(A)** RT–qPCR analysis of HMGA2 expression in four PDAC cell lines (BxPC-3, AsPC-1, PANC-1 and MIA PaCa-2). Transcript levels were normalized to GAPDH expression. **(B)** Western blot analysis of HMGA2 level in PANC-1 cells after transfecting NC and miR-93 mimic Cell protein was harvested 48h posttransfection and used for western blot analysis. **(C)** The binding region between wild-type HMGA2 3’UTR (base sequence in pale bule) and candidate microRNA (has-miR-93) (base sequence in red), and the mutated sites of HMGA2 3’UTR (base sequence in navy blue). **(D)** PANC-1 cells were co-transfected with miR-93 mimic and with either Wild-Type or MUT HMGA2 3’UTR reporter vector. Luciferase activities were tested 48h after transfection.

We examined TargetScan [22], miRanda, CLIP-Seq [23] and miRDB and found almost 100 potential miR-93 target genes. Of these candidate genes, HMGA2 was selected for its renowned EMT related function. First, we analysed the relative MIR-93 levels in all pancreatic cell lines (Figure 5A) and we choose PANC-1 as the tool cells for protein degradation experiment. Western blot of PANC-1 cells showed that miR-93 overexpression significantly decreased HMGA2 protein expression (Figure 5B). Next, we want to see whether the seed regions (Figure 5C) predicted by TargetScan actually have transcriptive activities. Reporter gene plasmids harboring either the 3’-UTR region wild-type HMGA2 or a 3’-UTR mutant (Figure 5C) and either miR-93 or NC were co-transfected into PANC-1. MiR-93 overexpression reduced luciferase activity compared with the reporter gene plasmid group containing the HMGA2 3’-UTR wildtype sequence (40% reduction) (Figure 5D).

### HMGA2 knock down suppress migration and invasion in pancreatic cell lines

**Figure 6:**
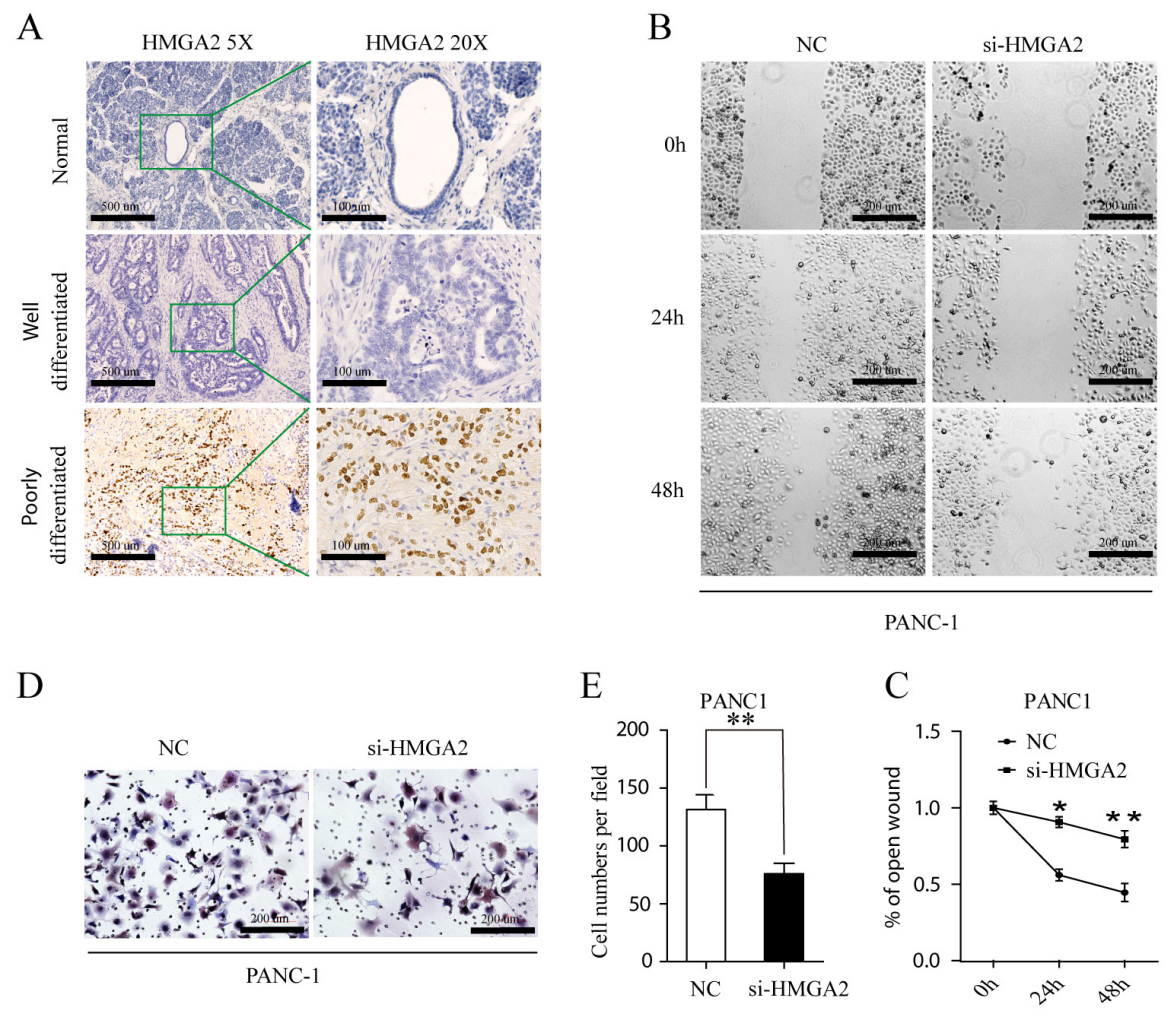
**(A)** Immunohistochemical staining for HMGA2 protein in normal, well- and poorly differnetiated PDAC (HMGA2 antibody, hematoxylin counterstain) with lower magnification images (5×, left) and its expanded views (20×, right). **(B, C)** Wound healing assay. PANC-1 cell was transfected with Negative Control (NC) and si-HMGA2. Optical images of wounded cell monolayers were taken after 0 h, 24 h and 48h. The remaining distance was calculated as a percentage of the initial wound area. The data presented were mean ± SEM of three independent experiments performed separately; ^*^P<0.05 or ^**^P<0.01. **(D, E)** PANC1 cells transfected NC and si-HMGA2 cells were induced to invade through Matrigel-coated transwell membranes. The number of migrated cells was counted. ^**^ P < 0.01.

*HMGA2* was up-regulated in poorly differentiated PDAC tissues (Figure 6A). We could reproduce the previously described *HMGA2* is overexpressed in high grade pancreatic ductal adenocarcinoma [24]. *HMGA2* was all negative in normal pancreatic tissues (Figure 6A). Besides, High *HMGA2* expression in tumor cells correlate with tumor differentiation P=0.007 (Supplementary Table 6). These indicated that *HMGA2* play important roles in tumor progression. Using wound healing cell migration assay and transwell cell invasion assay, the mobility of PANC-1 cells was significantly decreased following HMGA2 downregulation (Figure 6B–6C). Consistently, the invasive capacity was also drastically reduced following HMGA2 downregulation (Figure 6D–6E). MIA PaCa -2 cell line was also excluded from these assays owing to its low endogenous HMGA2 level, as showed in Figure 5A.

Quantitative PCR of miR-93 and HMGA2 were done in fresh pancreatic samples of cancers and normal tissues (Supplementary Figure 1), combining the opposite result of miR-93 and HMGA2 expression and function, we anticipated that miR-93 could work through regulating HMGA2 in PANC1 cells.

## DISCUSSION

In this study, we confirmed that the transcription factor, RUNX1, is upregulated in PDAC and plays many roles supporting tumor progression. In accordance with the microarray gene-expression (GSE71989 and GSE28735) results, RT–qPCR in fresh tissues also showed that RUNX1 levels were relatively higher in PDAC than in non-tumor pancreatic samples.

Using bioinformatics methods, we found that miR-93 is a potential target of RUNX1. MiR-93 does not have its own promoter and is localized in an intron of the MCM7 gene [25]. So an independent transcription factor can be an important regulator of miR-93 expression. This showed a possible TF-miRNA interaction rather than TF-gene interaction.

RUNX1 is a core transcription factor in hematopoiesis and functioning through specification and homeostasis of hematopoietic stem and progenitor cells (HSPCs) [26, 27], functioning as a DNA-binding transcription factor. RUNX1 can regulate miR27a, which itself also shown to regulate RUNX1, thereby forming a feed back loop during megakaryopoiesis [28]. MiR144/451 cluster can also be suppressed by RUNX1 through binding to the miR144/451 promotor, and RUNX1 controls a large number of hematopoietic related microRNAs during hematopoietic erythroid/megakaryocytic differentiation [29]. In gastric cancer, RUNX1 could reduce the aggressive function of miR-215, thus acting as a tumor suppressor [30]. In prostate cancer mouse model, RUNX-targeted miRNAs such as miR-23b, miR-205, miR-221 contribute to prostate cancer progression [31]. Here, we demonstrated that RUNX1 can regulate miR-93 in pancreatic cancers.

First, we identified a potential negative correlation of function between RUNX1 and miR-93 in pancreatic cells. RUNX1 overexpression promoted cell migration and invasion, while overexpression of miR-93 expression resulted in reduced cell migration and invasion. Overexpression of RUNX1 reduced miR-93 expression, whereas RUNX1 downregulation resulted in a marked increased miR-93 level, indicating that miR-93 is inhibited by RUNX1. But our relative expression of mRNA in pancreatic tissues showed a positive correlation between miR-93 and RUNX1, this may be due to lower amount of samples. MiR-93 expression in normal pancreas is higher than that in pancreatic cancer tissues.

Moreover, restoration of miR-93 expression results in suppressing EMT process. This phenomenon was more obvious in PANC-1 cells that have higher endogenous E-cadherin and HMGA2 expression; this also explains MIA PaCa -2 didn’t show the same behavior as pobble-like cell typed PANC-1. MiR-93 was once reported to slow cell proliferation and migration through direct degradation of p21 and CCNB1 in colon cancer, thus arresting the cell cycle in G2 stage [32]. In triple negative breast cancer cells *in vitro*, miR-93 overexpression also inhibit the invasive ability [33]. MiR-93 has also been found to inhibit EMT process of breast cancer cells through regulating MKL-1 and STAT3 [34]. Besides, miR-93 was showed to play important roles in promoting mesenchymal-epithelial (re-)transition (MET) in normal and malignant breast stem cells [35]. Despite these experimentally testified oncogenic protein-coding target genes regulated by miR-93, our study found that miR-93 also targets HMGA2.

HMGA2 was also an oncogene in tumorigenesis. HMGA2 expression was high in low differentiated pancreatic cancer and seldom in normal and high differentiated pancreatic cancer, suggesting an important role in tumor progression. HMGA2 also promoted the EMT process in pancreatic cancer [36]. This possible axis of RUNX1-miR-93-HMGA2 was never reported except for one article HMGA2 was directly regulated by RUNX1 during expansion of myeloid progenitors in mice in a cell type-dependent manner instead of binding to the HMGA2 promotor [37]. This study didn’t mention miR-93 and didn’t show TF-miRNA interaction or a TF-gene interaction. However, whether RUNX1 can diectly regulate the expression of HMGA2 in pancreatic cancer is still not clear, and we plan to testify this axis in the future *in vivo*.

Additionally, our data added deeper evidence that RUNX1 regulates miR-93 by directly binding to the DNA-binding sites in the miR-93 promotor. Our results demonstrated that in PANC-1, the far binding sites (Seq2) have more enrichment and in MIA PaCa -2 cells, the nearer binding sites (Seq1) have more enrichment. This may be due to the different phenotype of the two cells, which have different ancestors. As for the elevated transcriptive activity of RUNX1 overexpressed group verses empty group, testified through dual luciferase reporter assay, we thought this may be because of the different levels of endogenous RUNX1, MIA PaCa-2 cells has a higher endogenous RUNX1 level, but the transfection efficiency of pcDNA3.1-RUNX1 is not as high as that of PANC-1, so RUNX1 transfected group of PANC-1 didn’t show much of an increase in transcriptive activity than MIA PaCa -2 cells.

Our study also identified that increased RUNX1 level is responsible, at least in part, for the decreased level of miR-93, explaining the downregulation of miR-93 in pancreatic cancer tissues. MiR-93 is a new potential downstream pathway of RUNX1.

In summary, we identified that the transcription factor RUNX1 is overexpressed in PDAC tissues. RUNX1 promoted cell migration and invasion suggesting that it plays important roles in PDAC progression. RUNX1 directly regulated miR-93 through suppressing expression and function of miR-93, miR-93 functions as a suppress gene in pancreatic cancer and regulates its target such as HMGA2. This possible repression of miR-93 and HMGA2 interpreted TF-miRNA interaction and miRNA-gene interaction and provide us with insights into mechanisms underlying high levels of RUNX1 in PDAC progression. RUNX1 could be a promosing target in pancreatic cancer.

## MATERIALS AND METHODS

### Clinical samples and follow-up

We reviewed pancreatic surgical records of 2011 to 2013 in Peking Union Medical College Hospital and their hematoxylin-eosin-stained histological slices, and then selected 39 PDAC paraffin tissue blocks. All enrolled patients met the following inclusion criteria: pancreatic primary tumor; no chemotherapy or other treatments were given before surgery; resected samples were identified by pathological examination; pancreas cancer staging was determined in accordance with the 7^th^ edition of the American Joint Committee on Cancer pancreatic TNM classification [14]. Residual PDAC and adjacent non-tumor pancreatic tissues were collected immediately after pancreaticoduodenectomy (Whipple procedure) or pancreatectomy, and stored in liquid nitrogen. Clinical-pathological data of all cases enrolled in this study were listed in Supplementary Table 1. The Ethics Committee of Peking Union Medical College Hospital approved this study and all participants provided written informed consent.

Patients were followed up through checking medical records every 2 months during the first postoperative year and at least every 3∼4 months thereafter. Overall survival (OS) was defined as the time of surgery to death.

### Cell lines

Human pancreatic cancer cell lines PANC-1, MIA PaCa-2, AsPC-1 and BxPC-3 were aquired from American Type Culture Collection. PANC-1 and MIA PaCa-2 were cultured in DMEM containing 10% FBS, while AsPC-1 and BxPC-3 were cultured in RPMI with 10% FBS. All cells were maintained at 37°C in a humidified atmosphere with 5% CO_2_.

### Immunohistochemistry and scoring

All representive blocks were routinely fixed, embedded and sliced in paraffin. Immunohistochemical stainings were done using anti-RUNX1 (Cat. No. ab35962, polyclonal, dilution: 1:500, Abcam), and anti-HMGA2 (Cat. No. 8179, clone: D1A7, dilution: 1:400, Cell Signaling) antibodies, according to the manufacturers’ recommended staining protocols.

For HMGA2 and RUNX1, positive signals were located only in the nucleus. The results were analysed with a four-tiered system: 0 = negative; 1+ =< 10%; 2+ =10–50%; 3+ =>50% [11]. Tumors with scores of 0/1+ were regarded as low expression, and those scored 2+/3+ were regarded as high expression. All cases were independently scored by two pathologists (Yin Cheng and Shuangni Yu), with additional independent review by a third pathologist (Zhaohui Lu) in case of disagreement.

### RNA isolation, cDNA synthesis and qPCR

Total RNA of tissues or cell lines was extracted with TRizol (Life Technologies). For mRNA detection, first strand cDNA was synthesized from 1 μg total RNA templet using a High-Capacity cDNA Reverse Transcription Kit (Applied Biosystems). For the detection of miRNAs, 40 ng of total RNA templet was reverse transcribed using a TaqMan MicroRNA Reverse Transcription Kit (Applied Biosystems) with specific stem-loop reverse transcription (RT) primers (RiboBio, Guangzhou, China). cDNA was then 1:10 diluted for qPCR using SYBR green PCR master mix (Applied Biosystems) with specific primers. All real-time reactions were done in a 7500 Real Time PCR system (Applied Biosystems). GAPDH or U6 was used as an internal control. The sequences of primers used are listed in Supplementary Table 4.

### siRNA and miRNA transfection

For knocking down of RUNX1, two different siRNAs for depletion of RUNX1 were generated by Genepharma (Suzhou, Jiangsu, China). MiRNA mimics (for miR-93) were also synthesized and purified by Genepharma. Cells were transfected with 20 nM siRNAs or 50 nM miRNAs using the Lipofectamine RNAiMAX (Invitrogen) following manufacturer’s recommended protocol. The sequences of the RNA used in transfection are indicated in Supplementary Table 5.

### Plasmid construction and transfection

Full length of RUNX1 was inserted into the pcDNA3.1 (+) expression vector (Invitrogen) to overexpress this gene in pancreatic cancer cells. 3’ untranslated region of HMGA2 covering whole seed regions as well as mutate seed regions as controls were all cloned into pmirGLO (Promega), generating the plasmid pmirGLO-HMGA2-3’UTR WT or MUT. Seq1 and Seq1, Seq2 (sequence of miR-93 promoter containing RUNX1 binding sites) and Mut1, Mut2 (sequence of miR-93 promoter containing mutated RUNX1 binding sites) (Figure 3C) were cloned into pGL3-basic named as pGL3-miR93 Seq1-WT or pGL3-miR93 Seq1-Mut1. All Plasmids were verified by DNA sequencing. Lipofectamine 2000 Reagent (Invitrogen) was used for plasmids transfection.

### Cell migration and invasion assay

Wound-healing assay was used to assess cell migration ability. An artificial wound was created by 10μL pipette 72 hours posttransfection on the confluent cell layer. To observe the distance of cells migrated, photos were captured from the same area after 0, 24, 48, and 72 hours.

Invasion assay was completed by counting the number of cells transferring through Matrigel-coated membrane matrix insert (BD Biosciences). 2×10^5^cells suspended with serum free DMEM were seeded into an insert 72 hours posttransfection. The lower chamber contained DMEM with few FBS. 48 hours later, the inserts were fixed, stained with hematoxylin (Zhongshan) and counted.

### Western blot analysis

Whole cell lysate was lysised with RIPA buffer (Applygene Technologies Inc, Beijing, China) supplementing with Halt Protease Inhibitor Cocktail (Thermo Scientific, Rockford, USA). Protein concentration was calculated in accordance with a BCA reagent curve (Pierce Biotechnology, Rockford, IL). Equal amounts of protein (15 μg) were loaded on 8-12% SDS-PAGE gel and then transferred to PVDF membranes (Bio-Rad), which were incubated overnight at 4°C with primary antibodies against RUNX1 (1:1000), E-cadherin (1:400), Vimentin (1:1000), Snail (1:1000), Slug (1:2000), β-actin or GAPDH (1:1000), ZEB1 (1:400), K-ras (1:1000) or β-catenin (1:1000) respectively. The membranes were then incubated with the corresponding Peroxidase-linked secondary antibody for 1 h regularly, and the specific protein bands were visualized using ECL Western Blotting Substrate (Thermo Scientific).

### Luciferase assay

The promotor region of miR-93 covering the binding sites were each cloned in between KpnI and BagI of pGL3 basic vector (Promega). Mutated binding sites were created by PCR using Fast Mutagenesis System (Transgene). Cells with 80% confluence were transfected with pGL3-prom-miR93 (800ng per well), pRL-TK vector (200 ng per well) and pcDNA3.1 construct (RUNX1 or empty). PRL-TK vector serves as control. 48h later, luciferase activities were measured by Modulus^™^ II Detector (Sino Zhongyuan). The activity of each promoter was aquired by ratio of firefly to renilla.

### CHIP-qPCR assay

PANC-1, MIA PaCa-2 cells with 80% confluence were transfected with pcDNA3.1 construct (RUNX1 or empty) 2μg per well respectively.72h after transfection, cells were collected and ChIP was completed using an EZ-Magna ChIP^™^ kit (Millipore) according to the manufacturer’s recommended instructions. ChIP-grade antibodies for RUNX1 were obtained from Abcam (ab35962); Primers used for amplification of the regions including the potential RUNX1 binding sites in the promoter regions of miR-93 are listed in Supplementary Table 4.

### Statistical analysis

Statistical analyses were completed using IBM SPSS statistics V21 and GraphPad Prism 5 software. Student’s *t-test* was used to assess differences between groups. The difference of microRNA and gene expression between tumor and non-tumor tissues was compared using a MannWhitney *U-test*. Categorical variables were compared using Fisher’s exact test. Kaplan-Meier and log-rank tests were used for the cumulative survival analysis. Cox regression analysis was used for multivariate survival analysis. P < 0.05 was considered statistically significant.

## SUPPLEMENTARY MATERIALS FIGURE AND TABLES



## References

[1] Siegel RL, Miller KD, Jemal A (2016). Cancer statistics, 2016. CA Cancer J Clin.

[2] Ferlay J, Steliarova-Foucher E, Lortet-Tieulent J, Rosso S, Coebergh JW, Comber H, Forman D, Bray F (2013). Cancer incidence and mortality patterns in Europe: estimates for 40 countries in 2012. Eur J Cancer.

[3] Chen X, Yang H, Zhou X, Zhang L, Lu X (2016). MiR-93 Targeting EphA4 Promotes Neurite Outgrowth from Spinal Cord Neurons. J Mol Neurosci.

[4] Ryan DP, Hong TS, Bardeesy N (2014). Pancreatic adenocarcinoma. N Engl J Med.

[5] Kamisawa T, Wood LD, Itoi T, Takaori K Pancreatic cancer. The Lancet.

[6] Kayed H, Jiang X, Keleg S, Jesnowski R, Giese T, Berger MR, Esposito I, Lohr M, Friess H, Kleeff J (2007). Regulation and functional role of the Runt-related transcription factor-2 in pancreatic cancer. Br J Cancer.

[7] Whittle MC, Izeradjene K, Rani PG, Feng L, Carlson MA, DelGiorno KE, Wood LD, Goggins M, Hruban RH, Chang AE, Calses P, Thorsen SM, Hingorani SR (2015). RUNX3 Controls a Metastatic Switch in Pancreatic Ductal Adenocarcinoma. Cell.

[8] Whittle MC, Hingorani SR (2016). RUNX3 defines disease behavior in pancreatic ductal adenocarcinoma. Mol Cell Oncol.

[9] Banerji S, Cibulskis K, Rangel-Escareno C, Brown KK, Carter SL, Frederick AM, Lawrence MS, Sivachenko AY, Sougnez C, Zou L, Cortes ML, Fernandez-Lopez JC, Peng S (2012). Sequence analysis of mutations and translocations across breast cancer subtypes. Nature.

[10] Ellis MJ, Ding L, Shen D, Luo J, Suman VJ, Wallis JW, Van Tine BA, Hoog J, Goiffon RJ, Goldstein TC, Ng S, Lin L, Crowder R (2012). Whole-genome analysis informs breast cancer response to aromatase inhibition. Nature.

[11] Planaguma J, Diaz-Fuertes M, Gil-Moreno A, Abal M, Monge M, Garcia A, Baro T, Thomson TM, Xercavins J, Alameda F, Reventos J (2004). A differential gene expression profile reveals overexpression of RUNX1/AML1 in invasive endometrioid carcinoma. Cancer Res.

[12] Doll A, Gonzalez M, Abal M, Llaurado M, Rigau M, Colas E, Monge M, Xercavins J, Capella G, Diaz B, Gil-Moreno A, Alameda F, Reventos J (2009). An orthotopic endometrial cancer mouse model demonstrates a role for RUNX1 in distant metastasis. Int J Cancer.

[13] Wu J, Huang G, Ratner N (2015). Runx1: a new driver in neurofibromagenesis. Oncoscience.

[14] Vincent A, Herman J, Schulick R, Hruban RH, Goggins M (2011). Pancreatic cancer. Lancet.

[15] Yu S, Lu Z, Liu C, Meng Y, Ma Y, Zhao W, Liu J, Yu J, Chen J (2010). miRNA-96 suppresses KRAS and functions as a tumor suppressor gene in pancreatic cancer. Cancer Res.

[16] Zhao WG, Yu SN, Lu ZH, Ma YH, Gu YM, Chen J (2010). The miR-217 microRNA functions as a potential tumor suppressor in pancreatic ductal adenocarcinoma by targeting KRAS. Carcinogenesis.

[17] Jin X, Sun Y, Yang H, Li J, Yu S, Chang X, Lu Z, Chen J (2015). Deregulation of the MiR-193b-KRAS Axis Contributes to Impaired Cell Growth in Pancreatic Cancer. PLoS One.

[18] Wu Q, Qin H, Zhao Q, He XX (2015). Emerging role of transcription factor-microRNA-target gene feed-forward loops in cancer. Biomed Rep.

[19] Yang JH, Li JH, Jiang S, Zhou H, Qu LH (2013). ChIPBase: a database for decoding the transcriptional regulation of long non-coding RNA and microRNA genes from ChIP-Seq data. Nucleic Acids Res.

[20] Yevshin I, Sharipov R, Valeev T, Kel A, Kolpakov F (2017). GTRD: a database of transcription factor binding sites identified by ChIP-seq experiments. Nucleic Acids Res.

[21] Du L, Zhao Z, Ma X, Hsiao TH, Chen Y, Young E, Suraokar M, Wistuba I, Minna JD, Pertsemlidis A (2014). miR-93-directed downregulation of DAB2 defines a novel oncogenic pathway in lung cancer. Oncogene.

[22] Friedman RC, Farh KK, Burge CB, Bartel DP (2009). Most mammalian mRNAs are conserved targets of microRNAs. Genome Res.

[23] Chi SW, Zang JB, Mele A, Darnell RB (2009). Argonaute HITS-CLIP decodes microRNA-mRNA interaction maps. Nature.

[24] Hristov AC, Cope L, Reyes MD, Singh M, Iacobuzio-Donahue C, Maitra A, Resar LM (2009). HMGA2 protein expression correlates with lymph node metastasis and increased tumor grade in pancreatic ductal adenocarcinoma. Mod Pathol.

[25] Badal SS, Wang Y, Long J, Corcoran DL, Chang BH, Truong LD, Kanwar YS, Overbeek PA, Danesh FR (2016). miR-93 regulates Msk2-mediated chromatin remodelling in diabetic nephropathy. Nat Commun.

[26] Chen MJ, Yokomizo T, Zeigler BM, Dzierzak E, Speck NA (2009). Runx1 is required for the endothelial to haematopoietic cell transition but not thereafter. Nature.

[27] Kurokawa M (2006). AML1/Runx1 as a versatile regulator of hematopoiesis: regulation of its function and a role in adult hematopoiesis. Int J Hematol.

[28] Ben-Ami O, Pencovich N, Lotem J, Levanon D, Groner Y (2009). A regulatory interplay between miR-27a and Runx1 during megakaryopoiesis. Proc Natl Acad Sci U S A.

[29] Kohrs N, Kolodziej S, Kuvardina ON, Herglotz J, Yillah J, Herkt S, Piechatzek A, Salinas Riester G, Lingner T, Wichmann C, Bonig H, Seifried E, Platzbecker U (2016). MiR144/451 Expression Is Repressed by RUNX1 During Megakaryopoiesis and Disturbed by RUNX1/ETO. PLoS Genet.

[30] Li N, Zhang QY, Zou JL, Li ZW, Tian TT, Dong B, Liu XJ, Ge S, Zhu Y, Gao J, Shen L (2016). miR-215 promotes malignant progression of gastric cancer by targeting RUNX1. Oncotarget.

[31] Farina NH, Zingiryan A, Akech JA, Callahan CJ, Lu H, Stein JL, Languino LR, Stein GS, Lian JB (2016). A microRNA/Runx1/Runx2 network regulates prostate tumor progression from onset to adenocarcinoma in TRAMP mice. Oncotarget.

[32] Yang IP, Tsai HL, Hou MF, Chen KC, Tsai PC, Huang SW, Chou WW, Wang JY, Juo SH (2012). MicroRNA-93 inhibits tumor growth and early relapse of human colorectal cancer by affecting genes involved in the cell cycle. Carcinogenesis.

[33] Shyamasundar S, Lim JP, Bay BH (2016). miR-93 inhibits the invasive potential of triple-negative breast cancer cells *in vitro* via protein kinase WNK1. Int J Oncol.

[34] Xiang Y, Liao XH, Yu CX, Yao A, Qin H, Li JP, Hu P, Li H, Guo W, Gu CJ, Zhang TC (2017). MiR-93-5p inhibits the EMT of breast cancer cells via targeting MKL-1 and STAT3. Exp Cell Res.

[35] Liu S, Patel SH, Ginestier C, Ibarra I, Martin-Trevino R, Bai S, McDermott SP, Shang L, Ke J, Ou SJ, Heath A, Zhang KJ, Korkaya H (2012). MicroRNA93 regulates proliferation and differentiation of normal and malignant breast stem cells. PLoS Genet.

[36] Watanabe S, Ueda Y, Akaboshi S, Hino Y, Sekita Y, Nakao M (2009). HMGA2 maintains oncogenic RAS-induced epithelial-mesenchymal transition in human pancreatic cancer cells. Am J Pathol.

[37] Lam K, Muselman A, Du R, Harada Y, Scholl AG, Yan M, Matsuura S, Weng S, Harada H, Zhang DE (2014). Hmga2 is a direct target gene of RUNX1 and regulates expansion of myeloid progenitors in mice. Blood.

