# Supplementary material for: RUNX1 promote invasiveness in pancreatic ductal adenocarcinoma through regulating miR-93
Source: Oncotarget. 2017 Aug 24;8(59):99567–79. doi: 10.18632/oncotarget.20433 (PMC5725115; doi:10.18632/oncotarget.20433)
Supplement: Supplementary file 1 [file oncotarget-08-99567-s001.pdf]

RUNX1 promote invasiveness in pancreatic ductal adenocarcinoma through regulating miR-93

SUPPLEMENTARY MATERIALS

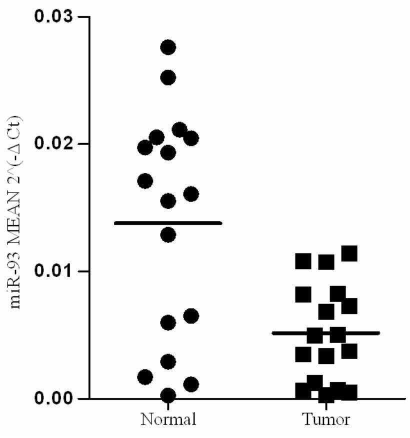

Supplementary Figure 1: Average relative miR-93 expression level in PDAC compared with that in normal tissues. Expression of miR-93 was measured by qRT-PCR and normalized by U6.

Supplementary Table 1: Clinical-pathological characteristics of 39 PDAC cases

Characteristics	Counts
**Gender** (male/female)	22/17
**Age** (<60 years/≥60 years)	19/20
**Tumor location** (head/ body-tail)	24/15
**Tumor differentiation** (Low/High-moderate)	14/25
**Tumor Size**(T1-2/T3-4)	3/36
**Lymph node metastasis** (Yes/No)	19/20
**Positive cutting margin**(Yes/No)	7/32
**TNM stage** (I- II/ III-IV)	31/8

Supplementary Table 2: Relation between clinical-pathological features and densities of RUNX1

Variables	RUNX1 (n=39)		P Value
	Low (n=13)	High (n=26)	
**Gender**			0.068
Female	3	14	
Male	10	12	
**Age**			0.821
≥60 years	7	13	
<60 years	6	13	
**Tumor location**			0.163
Body-tail	3	12	
Head	10	14	
**Lymph node metastasis**			0.365
Yes	5	14	
No	8	12	
**Poorly differentiation**			1.000*
Yes	5	9	
No	8	17	
**Positive cutting margin**			1.000*
Yes	2	5	
No	11	21	
**Tumor Size**			1.000*
T1-2	1	2	
T3-4	12	24	
**TNM stage**			0.888
I-II	11	20	
III/IV	2	6	

(*: Fisher's exact 2-tailed test).

Supplementary Table 3: Univariate and multivariate analyses of factors associated with survival

Variables	Overall Survival			
	Univariate	Mutivariate		
	P value	HR	95% CI	P Value
**Gender** (male/female)	0.013	0.688	0.499-5.573	0.407
**Age** (≤60 years / >60 years)	0.911	2.851	0.099-1.187	0.091
**Tumor location** (head/body-tail)	0.73	0.463	0.199-2.187	0.496
**Tumor differentiation** (I-II/III-IV)	0.458	1.334	0.145-1.647	0.248
**Positive cutting margin** (yes/no)	0.2	1.989	0.106-1.441	0.158
**Lymph node metastasis** (yes/no)	0.171	0.001	0.260-3.713	0.979
**Tumor Size** (T1-2/T3/4)	0.486	0.264	0.019-10.216	0.608
**TNM stage** (0, IA, IB / IIA, IIB / III, IV)	0.513	0.082	0.242-6.702	0.775
**RUNX1 expression**	0.127	3.177	0.083-1.126	0.075

Supplementary Table 4: Primers for RT-qPCR

Gene	Primer	Sequence
HMGA2	Forward primer	5'-ACCCAGGGGAAgACCCAA-3'
	Reverse primer	5'-CCTCTTGGCCGTTTTTCTCCA-3'
RUNX1	Forward primer	5'-CTCAGGTTTGTCGGTCGA-3'
	Reverse primer	5'-TGATGGCTCTGTGGTAGGTG-3'
GAPDH	Forward primer	5'-GCACCGTCAAGGCTGAGAAC-3'
	Reverse primer	5'-GCCTTCTCCATGGTGGTGAA-3'
miR-93	RT primer	5'-AAAGTGCTGTTCGTGCAGGTAG-3'
	Forward primer	5'-GCCGCAACTGGCCCTCAAAGT-3'
	Reverse primer	5'-GTGCAGGGTCCGAGGT-3'
U6	RT primer	5'-CGCTTCACGAATTTGCGTGTCAT-3'
	Forward primer	5'-GCTTCGGCAGCACATATACTAAAAT-3'
	Reverse primer	5'-CGCTTCACGAATTTGCGTGTCAT-3'
E-cadherin	Forward primer	5'-CTGAGAACGAGGCTAACG-3'
	Reverse primer	5'-GTCCACCATCATCATTCAATAT-3'
Vimentin	Forward primer	5'-TTGAACGCAAAGTGGAAT-3'
	Reverse primer	5'-AGGTCAGGCTTGGAAACA-3'
N-cadherin	Forward primer	5'-ATCCTACTGGACGGTTCG-3'
	Reverse primer	5'-TTGGCTAATGGCACTTGA-3'
Snail	Forward primer	5'-TCGCTGCCAATGCTCATC-3'
	Reverse primer	5'-AGCCTTTCCCACTGTCCTC-3'
Slug	Forward primer	5'-GACTACCGCTGCTCCATT-3'
	Reverse primer	5'-GAGGAGGTGTCAGATGGA-3'
Zeb1	Forward primer	5'-GATGATGAATGCGAGTCAGATGC-3'
	Reverse primer	5'-ACAGCAGTGTCTTGTTGTTGT-3'
β-catenin	Forward primer	5'-CCGATGCTGGGGACAAGAAT-3'
	Reverse primer	5'-CCCGTCATCCACCAAGACAC-3'
Seq1	Forward primer	5'-GACCAAGGTGCTTTTCTTCG-3'
	Reverse primer	5'-CTTGCATTGCTTGTGTATAGCAG-3'
Seq2	Forward primer	5'-GCAGAGTGGGGACTCACAGA-3'
	Reverse primer	5'-CTCTGGTCACACCCAGTCCT-3'

Supplementary Table 5: Sequences of siRNAs and miRNA mimics

Name		Nucleotide sequence
siRUNX1- I		5'-GCAGCUAAAUUACCAUAAAUU-3'
siRUNX1- II		5'-GGACCAGCCACAAACUUAAUU-3'
hsa-miR-93-mimic	Sense	5'-CAAAGUGCUGUUCGUGCAGGUAG-3'
	Antisense	5'-ACCUGCACGAACAGCACUUUGUU-3'
Negative control	Sense	5'-GUACCUGACUAGUCGCAGATT-3'
	Antisense	5'-UCUGCGACUAGUCAGGUACTT-3'
siHMGA2	Sense	5'-GCCGUCCACUUCAGCCCAGdTdT-3'
	Antisense	5'-CUGGGCUGAAGUGGACGGCdTdT -3'

Supplementary Table 6: Association of HMGA2 protein level with differentiation

	N	HMGA2		P value
		Positive	Negative	
Well-Moderately differentiated	23	22% (5)	78% (18)	0.007*
Poor differentiated	16	69% (11)	31% (5)	

(*: Fisher's exact 2-tailed test).
